# Predictive Effects of Inflammatory Scores in Patients with BCLC 0-A Hepatocellular Carcinoma after Hepatectomy

**DOI:** 10.3390/jcm8101676

**Published:** 2019-10-14

**Authors:** Pao-Yuan Huang, Chih-Chi Wang, Chih-Che Lin, Sheng-Nan Lu, Jing-Houng Wang, Chao-Hung Hung, Kwong-Ming Kee, Chien-Hung Chen, Kuang-Den Chen, Tsung-Hui Hu, Ming-Chao Tsai

**Affiliations:** 1 Division of Hepato-Gastroenterology, Department of Internal Medicine, Kaohsiung Chang Gung Memorial Hospital and Chang Gung University College of Medicine, Kaohsiung 83301, Taiwan; paoyuan813@gmail.com (P.-Y.H.); juten@ms17.hinet.net (S.-N.L.); jinghoung2001@yahoo.com.tw (J.-H.W.); chh4366@yahoo.com.tw (C.-H.H.); kee.kkm@gmail.com (K.-M.K.); e580306@ms31.hinet.net (C.-H.C.); dr.hu@msa.hinet.net (T.-H.H.); 2 Liver Transplantation Program and Department of Surgery, Kaohsiung Chang Gung Memorial Hospital, Chang Gung University College of Medicine, Kaohsiung 83301, Taiwan; ufel4996@gmail.com (C.-C.W.); immunologylin@gmail.com (C.-C.L.); 3 Division of Hepato-Gastroenterology, Department of Internal Medicine, Chiayi Chang Gung Memorial Hospital, Chiayi 61363, Taiwan; 4 Center for Translational Research in Biomedical Sciences, Liver Transplantation Program and Department of Surgery, Kaohsiung Chang Gung Memorial Hospital and Chang Gung University College of Medicine, Kaohsiung 83301, Taiwan; dennis8857@gmail.com; 5 Graduate Institute of Clinical Medical Sciences, Chang Gung University College of Medicine, Taoyuan 33302, Taiwan

**Keywords:** hepatocellular carcinoma, hepatectomy, inflammatory scores

## Abstract

Background: Inflammatory markers are regarded as prognostic factors of the outcomes of hepatocellular carcinoma (HCC). Examples include the neutrophil-to-lymphocyte ratio (NLR); platelet to lymphocyte ratio (PLR); the albumin and lymphocyte counts used in the prognostic nutritional index (PNI); and the neutrophil, lymphocyte, and platelet counts used in the systemic immune-inflammation index (SII). This study evaluates the effects of PNI, NLR, PLR, and SII to predict recurrence and survival in patients with Barcelona Clinic Liver Cancer (BCLC) stages 0-A of HCC after hepatectomy. Methods: This retrospective study was conducted at Kaohsiung Chung-Gung Memorial Hospital, Taiwan. The study enrolled 891 patients (77.9% males; mean age 58.53 ± 11.60 years) with BCLC stage 0/A HCC undergoing hepatectomy between 2001 and 2016. PNI, NLR, PLR and SII were measured before hepatectomy. Results: High NLR (>1.8) was adversely associated with overall survival (*p* = 0.032). Low PNI (≤45) was adversely associated with overall survival and disease-free survival (*p* < 0.001). Low SII (≤45) also had an adverse association with overall survival (*p* = 0.008) and disease-free survival (*p* < 0.001). Diabetes mellitus, cirrhosis, microvascular invasion, low PNI (≤45), and low SII (≤160) were independently associated with poor overall survival in a multivariate analysis. HCV infection, diabetes mellitus, cirrhosis, microvascular invasion, low PNI, and low SII were independent prognostic factors of recurrent HCC. The combined use of PNI and SII provided improved prognostic information. Conclusions: Low PNI and low SII are significantly poor prognostic factors for overall survival and recurrence in patients with BCLC 0-A hepatocellular carcinoma after hepatectomy.

## 1. Introduction

Liver cancer is the sixth most commonly diagnosed cancer and the fourth leading cause of cancer death worldwide as of 2018. Hepatocellular carcinoma (HCC) comprises 75–85% of primary liver cancer cases [1]. The management of HCC depends on the tumor stage at diagnosis and liver function. Current management strategies involve resection, liver transplantation (LT), radiofrequency ablation (RFA), transarterial embolization (TAE)/transarterial chemoembolization (TACE), radiation therapy, or systemic therapy [2,3]. Patients are considered to have resectable tumors if they have one to three unilobar lesions with an upper limit of 5 cm for a single lesion and 3 cm for more than one lesion; no extrahepatic metastasis or macrovascular invasion; and little or no portal hypertension [2].

Hepatectomy is a potentially curative treatment for HCC, but the long-term prognosis remains unsatisfactory due to the high incidence of recurrence (50–60%) [4,5,6]. Prognostic factors for recurrence and survival after resection include tumor size, α-fetoprotein level, tumor differentiation, microvascular invasion, cirrhosis, surgical margin, and metabolic syndrome [6,7,8]. Recently, the associations between inflammatory markers and the prognosis of HCC have been actively explored, and systemic inflammation has been thought to be related to poor outcomes. The reason is that inflammatory responses play critical roles in tumorigenesis, including initiation, promotion, angiogenesis, invasion, and metastasis [9], and an inflammatory microenvironment is an important condition for tumors [10].

Systemic inflammatory responses have been investigated using markers such as the neutrophil-to-lymphocyte ratio (NLR) [11,12,13,14], platelet to lymphocyte ratio (PLR) [15,16,17], albumin and lymphocyte counts used in the prognostic nutritional index (PNI) [18,19,20], and neutrophil, lymphocyte, and platelet counts used in the systemic immune-inflammation index (SII) [21,22]. To date, most studies have evaluated only one of the inflammatory parameters. Therefore, a comprehensive evaluation of the prognostic values of the inflammatory markers would be valuable for patients with HCC. The aim of this study is to evaluate the effects of inflammatory markers to predict recurrence and survival in HCC patients who have Barcelona Clinic Liver Cancer (BCLC) grades 0–A and are receiving hepatectomy.

## 2. Materials and Methods

### 2.1. Ethics Statement

The study protocol was approved by the institutional review board and the ethics committee of Chang Gung Memorial Hospital (201901103B0). The ethics committee waived the requirement for informed consent for this study, and all the data were analyzed anonymously.

### 2.2. Data Sources

**Figure 1:**
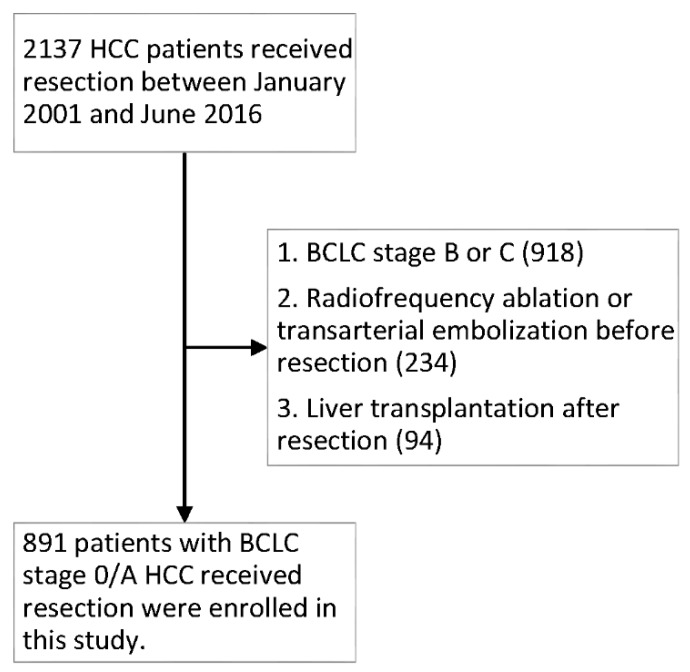
Flow chart of patients included in this study. HCC, hepatocellular carcinoma; BCLC, Barcelona Clinic Liver Cancer.

This retrospective study enrolled patients with newly diagnosed HCC who underwent surgical intervention between January 2001 and June 2016 at Kaohsiung Chung-Gung Memorial Hospital. HCC was diagnosed based on histological evidence or classical imaging features according to the guidelines of the American Association for the Study of the Liver [23]. The staging of tumors was determined according to the BCLC staging classification [24]. Patients who had RFA or TAE before resection, LT after resection, or BCLC stage B or C were excluded from the study (Figure 1). Surgical resection for tumors in BCLC stage 0 or A is the widely accepted standard treatment. However, surgical treatment for BCLC stage B or C HCC remains controversial. Recently, the management of HCC has tended to involve a multidisciplinary approach, such as RFA or TACE combined with surgery, etc. Because RFA and TACE are associated with the induction of inflammation and immunological responses, it might have some degree of influence on inflammatory indicators. Hence, only treatment-naïve HCC patients were enrolled in our study. LT was believed to cure both the tumor and the underlying cirrhosis at the same time in well-selected patients, which also have an influence on the survival. Therefore, those who received salvage LT were excluded [25].

### 2.3. Methods

Patient demographics and clinical characteristics were obtained from the reviewed medical records, including complete blood counts, albumin, α-fetoprotein level, Child–Turcotte–Pugh (CTP) class, date of diagnosis, status of viral hepatitis, tumor staging by BCLC, duration of follow-up, and outcomes. Blood tests were taken within 1 week before operation. NLR was calculated by dividing the absolute neutrophil count by the lymphocyte count, and PLR was obtained by dividing the platelet count by the lymphocyte count. PNI was calculated using the following equation: lymphocyte count (/μL) × 0.005 + albumin (g/dL) × 10. SII was measured as platelet count × neutrophil count/lymphocyte count (/μL) [26]. Recurrence-free survival (RFS) was defined as the period from tumor removal by resection until the detection of recurrent or metastatic disease. Overall survival (OS) was defined as the time from the diagnosis to death, last contact, or 28 February 2017.

### 2.4. Statistical Analysis

Descriptive analyses were used to determine the patients’ clinicopathological characteristics at baseline. Kaplan–Meier survival curves were constructed for each variable. A Cox proportional hazard model was used for the multivariate analysis of the hazard ratio (HR). All statistical analyses were conducted using software SPSS Version 23.0. (IBM Corp., Armonk, NY, USA) A two-sided *p* value < 0.05 was considered significant.

## 3. Results

### 3.1. Clinical Characteristics

A total of 2137 patients received hepatectomy for HCC between January 2001 and June 2016. Overall, 1246 patients were excluded due to intermediate to advanced stages (BCLC stage B or C) (*n* = 918), RFA or TAE before operation (*n* = 234), and liver transplantation after resection (*n* = 94). The remaining 891 patients were eligible for the analysis (Figure 1).

The mean age was 58.53 years, and the majority of patients were men (*n* = 694, 77.9%). There were 239 patients (27.8%) who were diabetic before surgery, and 417 patients (46.8%) were diagnosed with cirrhosis. Cirrhosis was defined as METAVIR stage 4 fibrosis based on reports of the histopathological evaluation of non-tumor liver tissue obtained by surgery at the time of the resection of the tumor [27]. Most patients were classified as CTP A (*n* = 819, 91.9%). The median follow-up time was 5.30 ± 3.04 years, and a total of 433 (48.6%) patients had recurrent disease during follow-up (Table 1).

### 3.2. Optimal Cutoff Values for Inflammatory Markers

**Figure 2:**
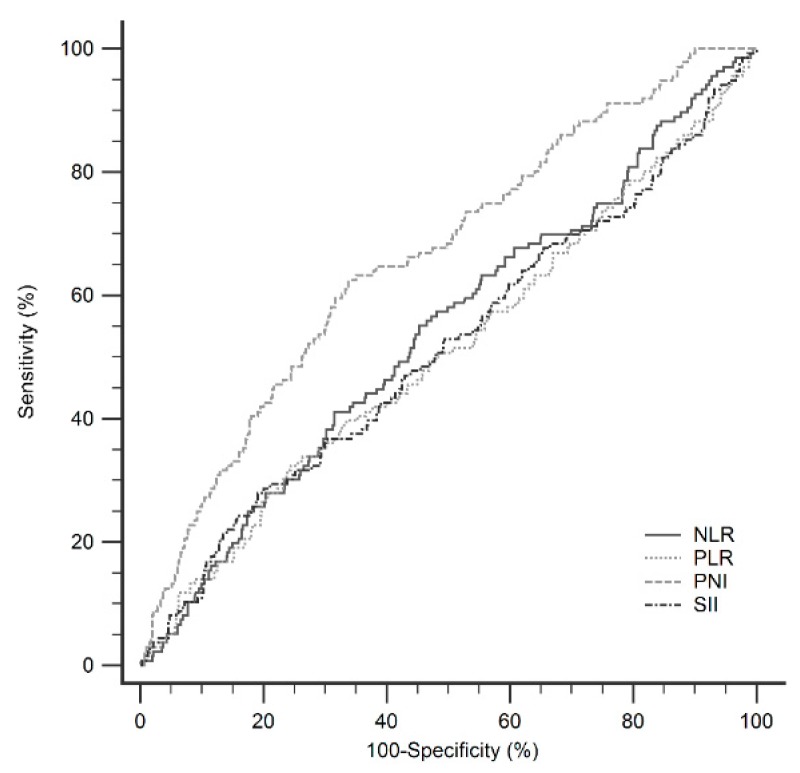
Comparisons of the areas under the curve (AUC) for outcome predictions in patients after operations.

Each inflammatory marker was stratified according the maximum sensitivity and specificity using Youden’s index. The optimal cutoff value for NLR was 1.8 (AUROC curve: 0.539; 95% confidence interval (CI): 0.501–0.577). The optimal cutoff value for PLR was 116.6 (AUROC curve: 0.509; 95% CI: 0.471–0.546). The optimal cutoff value for PNI was 42.8 (AUROC curve: 0.663; 95% CI: 0.626–0.698). The optimal cutoff value for SII was 160.0 (AUROC curve: 0.514; 95% CI: 0.476–0.551) (Table 2 and Figure 2).

### 3.3. Survival Analysis

**Figure 3:**
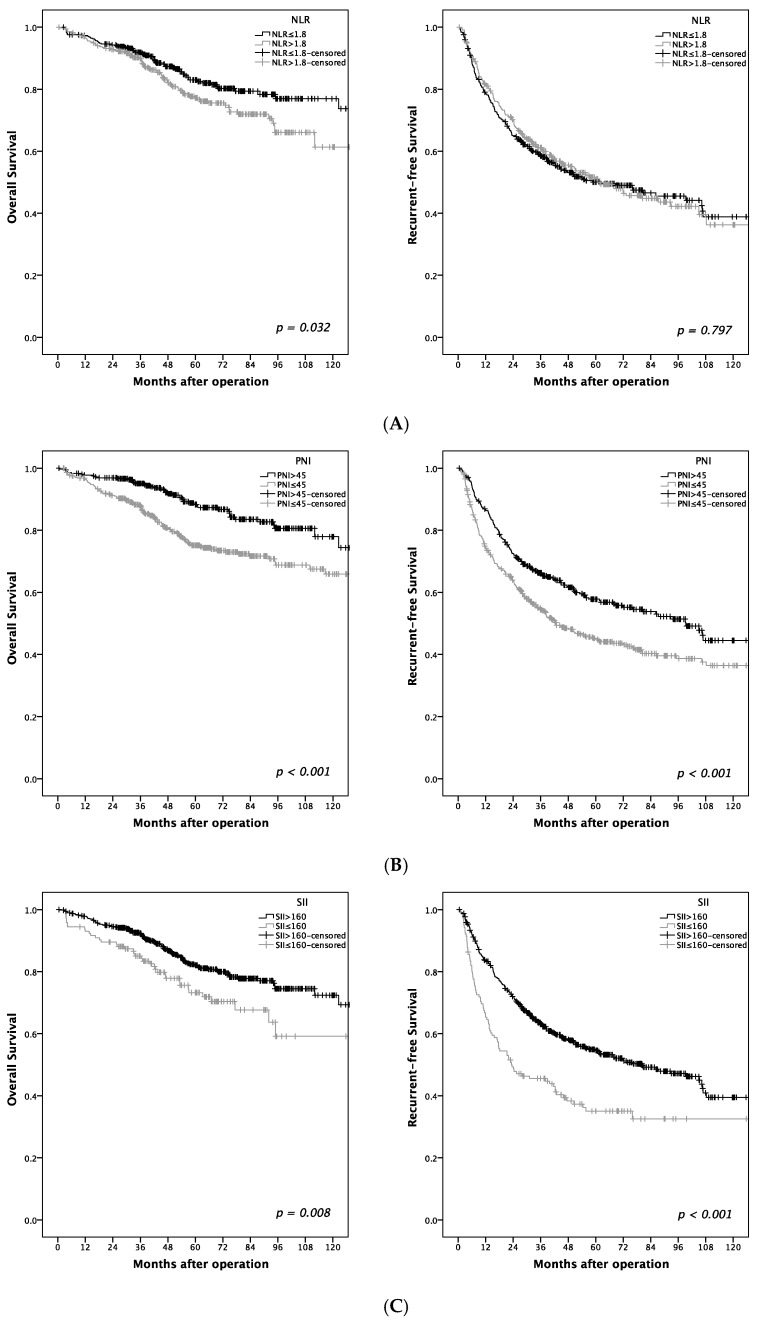
Relationship of (**A**) neutrophil-to-lymphocyte ratio (NLR), (**B**) prognostic nutrition index (PNI), and (**C**) systemic Immune-Inflammation Index (SII) to overall survival (OS), as well as recurrent free survival (RFS).

**Figure 4:**
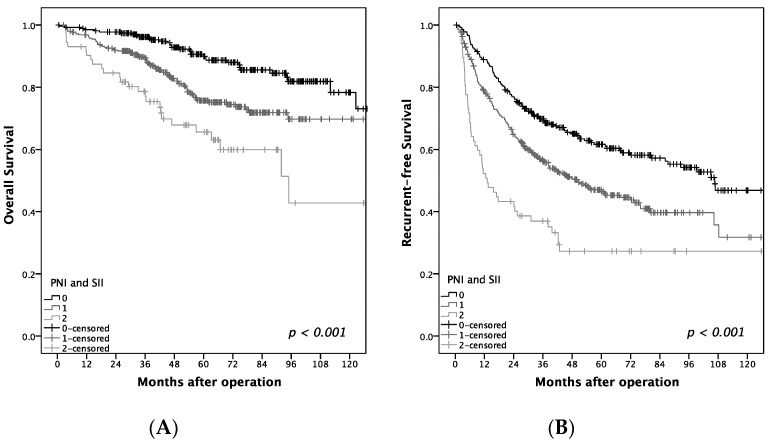
Relationship of prognostic nutrition index (PNI) and systemic Immune-Inflammation Index (SII) to (**A**) overall survival (OS) and (**B**) recurrence-free survival. Group 0 = PNI > 45 and SII > 160, group 1 = PNI > 45/SII ≤ 160 or PNI ≤ 45/SII > 160, group 2 = PNI ≤ 45 and SII ≤ 160.

The one- and five-year OS rates were 97 and 82%, respectively. As shown in Table 3, old age (>60 years old; *p* = 0.024), diabetes mellitus (*p* < 0.001), cirrhosis (*p* < 0.001), microvascular invasion (*p* < 0.001), high AFP (> 5 μg/L; *p* = 0.047), high NLR (>1.8; *p* = 0.032), low PNI (≤45; *p* < 0.001), and low SII (≤160; *p* = 0.008) were associated with high OS in BCLC 0-A HCC patients after hepatectomy. The relationships of NLR, PNI, and SII with OS and RFS are shown in Figure 3. High NLR was significantly associated with poor OS (*p* = 0.032). However, high PNI was significantly associated with better OS (*p* < 0.001) and RFS (*p* < 0.001). In addition, elevated SII was associated with better OS (*p* = 0.008) and RFS (*p* < 0.001). The one-year, three-year, and five-year OS rates of patients with PNI > 45 and SII > 160 were 99%, 96%, and 90% respectively, which were significantly higher than those observed with PNI ≤ 45 and SII ≤ 160 (90%, 77%, and 66%) (Figure 4).

In the univariate analysis, old age (>60 years old), HCV infection, HBV and HCV coinfection, diabetes mellitus, cirrhosis, microvascular infection, high AFP (>5 μg/L), high NLR (>1.8), low PNI (≤45), and low SII (160 × 10^9^/L) were associated with poor OS. The multivariate analysis of these parameters revealed that diabetes mellitus, cirrhosis, microvascular infection, low PNI (≤45), and low SII (≤160 × 10^9^/L) were independently associated with poor OS. The HR of death for low PNI was 1.789 (95% CI 1.217–2.630, *p* = 0.003), and that for low SII was 1.674 (95% CI 1.097–2.552, *p* = 0.017) (Table 4).

Overall, 458 patients (51.4%) were free from tumor relapse during the follow-up period, and the mean time to recurrence was 3.80 ± 3.17 years. The one- and five-year RFS rates were 80 and 51%, respectively. The univariate analysis showed that poor RFS was also associated with old age (>60 years old), HCV infection, HBV and HCV coinfection, diabetes mellitus, cirrhosis, microvascular invasion, high AFP (> 5 μg/L), low PNI (≤45), and SII (≤160 × 10^9^/L). The multivariate analysis demonstrated that HCV infection, diabetes mellitus, cirrhosis, microvascular invasion, low PNI (≤45), and low SII (≤160 × 10^9^/L) were independent prognostic factors for RFS. The HR of tumor recurrence for low PNI was 1.378 (95% CI 1.092–1.738, *p* = 0.007), and that for low SII was 1.616 (95% CI 1.231–2.123, *p* = 0.001) (Table 5).

The relationship between inflammatory markers and clinicopathological features is summarized in Table 6; Table 7. Low PNI (≤45) was associated with old age (*p* < 0.001), HCV infection (*p* < 0.001), raised alanine aminotransferase (ALT) (*p* < 0.001), decreased albumin (*p* < 0.001), and raised INR (*p* < 0.001). SII ≤ 160 was associated with raised INR (*p* < 0.001) and microvascular invasion (*p* = 0.034).

## 4. Discussion

Surgical resection is the mainstay of curative treatment for very-early and early-stage HCC with preserved liver function. Tumor recurrence is still the main concern of resection, and the five-year cumulative HCC recurrent rates are more than 50% [2,28]. Many studies have shown the relationships between inflammatory response and the development of tumors, and several scoring systems have been developed to predict the prognosis in cancer patients. NLR, PLR, PNI, and SII have been reported to be useful in predicting OS and recurrence in HCC patients.

We investigated the clinical and prognostic value of these widely used inflammatory markers in early-stage HCC patients receiving operation and compared their predictive accuracy. Our retrospective cohort of 891 patients revealed that PNI and SII are independent predictors of OS and tumor recurrence. Furthermore, the predictive power of the PNI score outweighs that of other inflammatory markers. PNI and SII are derived from routinely available blood tests, making them easy to apply in routine clinical practice to predict the prognosis in HCC patients.

Studies have shown that the systemic inflammatory response as measured by the NLR, PLR, and SII are good predictors of tumor outcomes. The exact mechanism remains unclear, but several hypotheses have been proposed. Basic studies have revealed that neutrophils can induce tumor proliferation and angiogenesis, as well as enhance the migration and metastasis of cancer cells. In addition, HCC cells induce neutrophils to release hepatocyte growth factor, which makes cancer cells become more aggressive [29]. Platelets can be activated by immune cells and cancer cells and play an important role in tumor metastasis, tumor growth, and angiogenesis. This occurs by protecting cancer cells from immune surveillance, facilitating cancer cell arrest within the vasculature and subsequent tissue invasion, inducing endothelial cell proliferation and new blood vessel formation, and interacting with cancer cells and stroma in the tumor microenvironment [30]. However, lymphocytes, including T cells, B cells, and natural killer cells, have antitumor effects either directly or upon the activation of other lymphocytes [31].

Higher levels of NLR and PLR are associated with poor outcomes in many types of cancer. In a meta-analysis of patients with HCC, Zeng et al. examined 24 studies and concluded that a high NLR predicted a poor OS (HR: 1.54, 95% CI 1.34–1.76, *p* < 0.001) and poor RFS (HR: 1.45, 95% CI 1.16–1.82, *p* = 0.001), while a high PLR predicted poor OS (HR: 1.63, 95% CI 1.34–1.98, *p* < 0.001) and poor RFS (HR: 1.52, 95% CI 1.21–1.91, *p* < 0.001) [32]. In the present study, a high NLR (>1.8) before surgical resection predicted poor OS in HCC patients, but NLR was not an independent factor in predicting OS and RFS in the multivariate analyses. PLR had no significant association with OS or RFS.

Higher SII has been reported to be a poor prognostic factor in multiple cancers. A recent meta-analysis included 22 studies with 7657 patients and showed that a higher level of SII was correlated with poor OS (HR: 1.69, 95% CI 1.42–2.01, *p* < 0.001) and poor RFS (HR = 1.66, *p* = 0.025) in patients with cancers. Furthermore, a subgroup analysis revealed that higher SII than a cutoff value could predict poor OS in HCC (*p* < 0.001) [33]. However, our study revealed the opposite result: low SII predicted a poor prognosis of OS (HR: 1.674, 95% CI 1.097–2.552, *p* = 0.017) and RFS (HR: 1.616, 95% CI: 1.231–2.123, *p* = 0.001). Lower SII means lower neutrophil levels, lower platelet levels, and higher lymphocyte levels, as shown in Table 7. Thrombocytopenia was an independent predictor for survival in patients with compensated cirrhosis and HCC treated with hepatectomy [34]. Through a meta-analysis, Zhang et al. showed that thrombocytopenia in HCC patients was associated with poor OS (HR: 1.47, 95% CI 1.21–1.78) and poor RFS (HR: 1.41, 95% CI: 1.22–1.62) in HCC patients after hepatic resection [35]. Platelet count has been used to assess the severity of chronic liver disease and features of portal hypertension [36]. In the present study, low SII was associated with high APRI and high INR, which are related to significant fibrosis and poor liver function. In the subgroup analysis stratified by platelet count, low SII in the thrombocytopenia group revealed significantly poor OS (*p* = 0.019) and RFS (*p* = 0.002) compared to those with high SII, but there was no statistical significance in the non-thrombocytopenia group. Therefore, low SII is associated with poor outcome due to advanced liver disease, especially in those with thrombocytopenia.

The PNI was initially designed to assess the immunological and nutritional condition of patients undergoing surgery for digestive diseases [37]. It was first used to predict HCC outcome as an inflammatory marker by Pinato et al. in 2012 [18]. Lymphopenia is an unfavorable prognostic factor for OS in several cancers [38], which may result from an increased rate of turnover of lymphocytes induced by tumor cells [39] or an altered homeostasis of lymphocytes in patients with tumors [40]. Albumin synthesis is reduced by the systemic inflammatory response to a tumor [41], although impaired hepatic synthetic function in advanced liver disease also needs to be considered as an additional cause for reduced serum albumin. Chan et al. reported that the PNI was a significant predictor for OS and RFS in patients with very-early/early-stage HCC receiving curative surgery [20]. Our data are consistent with previous studies suggesting that low PNI predicts poor OS and RFS in HCC patients receiving hepatectomy. We subsequently compared the predictive power of the PNI with other inflammatory markers, including NLR, PLR, and SII, and the PNI produced superior results.

Nevertheless, there are some limitations to our study. First, it is a retrospective study with patients from a single institution, which could lead to biases. Second, more than half of the patients in the study had HBV infection as the etiology of HCC, whereas chronic HCV infection is the major cause for the development of HCC in Western countries and Japan [42]. Moreover, laboratory values were only collected before surgery, and a serial follow-up of inflammatory markers after surgery may provide more insights in the prognostic values.

In conclusion, among the evaluated preoperative serum inflammatory markers, low PNI and low SII were independently associated with unfavorable outcomes of OS and RFS in patients with BCLC 0-A hepatocellular carcinoma after hepatectomy. Those observed with low PNI or low SII, especially PNI ≤ 45 and SII ≤ 160 with a high one-year recurrence rate (50%), need a close follow-up, and further adjuvant therapies, such as target or immune-based therapy, might be required. However, the side effects of drugs should be watched for, and additional well-designed studies are needed to confirm this concept. Further evaluation of these markers should be performed on a larger scale and compared between different patient populations.

## Figures and Tables

**Table 1:** Patients’ baseline characteristics before hepatectomy.

Number of Patients	891
Median follow-up (Years)	5.30 ± 3.04
Mean Age (Years)	58.54 ± 11.60
HBV Infection. *n* (%)	502 (56.3)
HCV Infection. *n* (%)	311 (34.9)
HBV + HCV Coinfection. *n* (%)	44 (4.9)
Male. *n* (%)	694 (77.9)
Diabetes Mellitus. *n* (%)	239 (27.8)
AFP > 5 ng/mL. *n* (%)	601 (67.5)
Cirrhosis. *n* (%)	417 (46.8)
Child–Turcotte–Pugh. *n* (%)	
A	819 (91.9)
B	71 (8)
BCLC Stage. *n* (%)	
0	125 (14)
A	766 (86)
Tumor Differentiation. *n* (%)	
Well	111 (12.5)
Moderate	743 (83.4)
Poor	24 (2.7)
HCC Recurrence. *n* (%)	433 (48.6)
Mean time to Recurrence (years)	3.80 ± 3.17
Mean NLR Before Surgery	2.81 ± 3.99
NLR > 1.8 before Surgery. *n* (%)	338 (47.7)
Mean PLR before Surgery	102.14 ± 67.97
PLR > 120 before Surgery. *n* (%)	178 (25.2)
Mean PNI before Surgery	44.18 ± 7.38
PNI ≤ 45 before Surgery. *n* (%)	441 (55.1)
Mean SII before Surgery (×10^9^/L)	442.23 ± 680.78
SII ≤ 160 before Surgery. *n* (%)	144 (20.5)

Data are expressed as mean ± standard deviation or number (percentage). Abbreviations: HBV: hepatitis B virus; HCV: hepatitis C virus; AFP: alpha-fetoprotein; BCLC stage: Barcelona clinic liver cancer stage; HCC: hepatocellular carcinoma; NLR: neutrophil-to-lymphocyte ratio; PLR: platelet to lymphocyte ratio; PNI: prognostic nutritional index; SII: systemic immune-inflammation index.

**Table 2:** Comparisons of the areas under the curve (AUC) values and the cutoff values among the inflammatory markers.

	AUC	CUTOFF	SE	95% CI
**NLR**	0.539	1.809	0.028	0.501–0.577
**PLR**	0.509	116.618	0.029	0.471–0.546
**PNI**	0.663	42.844	0.026	0.626–0.698
**SII**	0.514	159.984	0.029	0.476–0.551

Neutrophil-to-lymphocyte ratio (NLR) = Neutrophil/lymphocyte, platelet to lymphocyte ratio (PLR) = platelet/lymphocyte, Prognostic nutritional index (PNI) = lymphocyte(/μL) × 0.005 + albumin (g/dL) × 10, Systemic Immune-Inflammation Index (SII) = Neutrophil × platelet/lymphocyte.

**Table 3:** Cumulative survival rates in BCLC 0-A HCC patients after hepatectomy.

Variables	Patient No.	1 Year (%)	3 Years (%)	5 Years (%)	*p* Value
Age (Years)					
>60	464	96.7	89.2	78.2	0.024
≤60	427	97.6	93.9	85.5	
Gender					
Male	694	96.9	92.0	82.4	0.792
Female	197	98.0	89.3	79.4	
Diabetes Mellitus					
Yes	239	97.0	88.8	72.3	<0.001
No	621	97.1	92.5	84.9	
Liver Cirrhosis					
Yes	417	95.7	87.5	75.9	<0.001
No	474	98.5	94.9	86.8	
Differentiation					
Moderate or Poor	767	97.0	91.3	81.2	0.201
Well	111	98.2	91.4	84.8	
Microvascular Invasion					
Yes	330	94.5	87,2	75.5	<0.001
No	561	98.7	94.0	85.3	
Serum AFP (μg/L)					
>5	601	96.5	90.5	80.3	0.047
≤5	234	98.7	95.0	86.9	
NLR					
>1.8	338	96.4	89.8	77.2	0.032
≤1.8	370	97.3	91.5	83.0	
PLR					
>120	178	96.1	89.2	75.7	0.169
≤120	529	97.1	91.2	81.8	
PNI					
>45	359	97.8	95.0	88.2	<0.001
≤45	441	96.3	87.6	75.2	
SII (×10^9^/L)					
>160	559	97.8	92.3	82.1	0.008
≤160	144	93.0	84.2	73.2	

Abbreviations: HCC: hepatocellular carcinoma; AFP: alpha-fetoprotein; NLR: neutrophil-to-lymphocyte ratio; PLR: platelet to lymphocyte ratio; PNI: prognostic nutritional index; SII: systemic immune-inflammation index.

**Table 4:** Uni-and multivariate analyses of factors associated with mortality by Cox proportional hazards model.

	Univariate			Multivariate		
	HR	95% CI	*p* Value	HR	95% CI	*p* Value
Male Sex	1.050	0.728–1.516	0.793			
Age (>60 Years)	1.601	1.173–2.187	0.003			
HBV Infection	0.922	0.562–1.514	0.794			
HCV Infection	1.347	0.810–2.240	0.251			
HBV + HCV Coinfection	1.532	0.748–3.134	0.243			
Diabetes Mellitus	2.066	1.499–2.848	<0.001	1.989	1.362–2.906	<0.001
Cirrhosis	1.978	1.442–2.713	<0.001	1.502	1.013–2.227	0.043
Moderate/poor Differentiation	1.367	0.836–2.235	0.213			
Microvascular Invasion	2.040	1.427–2.915	<0.001	1.642	1.123–2.401	0.011
AFP > 5 μg/L	1.541	1.044–2.276	0.030			
NLR > 1.8	1.441	1.029–1.071	0.033			
PLR > 120	1.287	0.898–1.845	0.170			
PNI ≤ 45	1.976	1.401–2.788	<0.001	1.789	1.217–2.630	0.003
SII ≤ 160 (×10^9^/L)	1.654	1.137–2.405	0.008	1.674	1.097–2.552	0.017

Abbreviations: HR: hazard ratio; CI: confidence interval; HBV: hepatitis B virus; HCV: hepatitis C virus; AFP: alpha-fetoprotein; NLR: neutrophil-to-lymphocyte ratio; PLR: platelet to lymphocyte ratio; PNI: prognostic nutritional index; SII: systemic immune-inflammation index.

**Table 5:** Uni- and multivariate analyses of factors associated with recurrence by Cox proportional hazards model.

	Univariate			Multivariate		
	HR	95% CI	*p* Value	HR	95% CI	*p* Value
Male Sex	0.978	0.779–1.228	0.848			
Age (>60 Years)	1.382	1.144–1.670	0.001			
HBV Infection	1.214	0.885–1.665	0.230			
HCV Infection	1.651	1.189–2.293	0.003	1.298	1.022–1.649	0.033
HBV + HCV Coinfection	1.880	1.164–3.036	0.010			
Diabetes Mellitus	1.368	1.110–1.687	0.003	1.352	1.05–1.734	0.017
Cirrhosis	1.762	1.457–2.132	<0.001	1.536	1.21–1.950	<0.001
Moderate/Poor Differentiation	1.005	0.760–1.329	0.972			
Microvascular Invasion	1.653	1.325–2.062	<0.001	1.542	1.218–1.951	<0.001
AFP > 5 μg/L	1.398	1.116–1.752	0.004			
NLR > 1.8	0.972	0.786–1.203	0.797			
PLR > 120	0.975	0.765–1.243	0.839			
PNI ≤ 45	1.462	1.195–1.790	<0.001	1.378	1.092–1.738	0.007
SII ≤ 160 (×10^9^/L)	1.751	1.372–2.234	<0.001	1.616	1.231–2.123	0.001

Abbreviations: HR: hazard ratio; CI: confidence interval; HBV: hepatitis B virus; HCV: hepatitis C virus; AFP: alpha-fetoprotein; NLR: neutrophil-to-lymphocyte ratio; PLR: platelet to lymphocyte ratio; PNI: prognostic nutritional index; SII: systemic immune-inflammation index.

**Table 6:** Comparison of characteristics between patients with high and low PNI.

	High PNI (>45, *n* = 362)	Low PNI (≤45, *n* = 444)	*p* Value
Male Sex	294 (81.2%)	338 (76.1%)	0.086
Age, Years	55.67 ± 12.23	60.83 ± 10.79	<0.001
HBV Infection	228 (63.0%)	230 (51.8%)	0.002
HCV Infection	95 (26.2%)	187 (42.1%)	<0.001
APRI	0.61 ± 0.47	1.24 ± 2.01	<0.001
ALT, U/L	42.24 ± 26.79	63.61 ± 103.31	<0.001
Neutrophil, /μL	3240.26 ± 1210.93	4181.64 ± 3668.09	<0.001
Lymphocyte, /μL	2109.12 ± 641.08	1445.07 ± 560.36	<0.001
Platelet, ×10^3^/μL	170.90 ± 55.62	151.95 ± 89.63	0.001
Total Bilirubin, mg/dL	0.80 ± 0.30	0.83 ± 0.36	0.171
Albumin, g/dL	4.07 ± 0.40	3.30 ± 0.54	<0.001
Prothrombin Time, INR	1.02 ± 0.056	1.05 ± 0.96	<0.001
Moderate/Poor Differentiation	314 (89.0%)	376 (85.6%)	0.200
Microvascular Invasion	130 (46.4%)	158 (48.9%)	0.568
AFP, μg/L	416.47 ± 1620.65	661.87 ± 3242.02	0.195

Data are expressed as mean ± standard deviation or number (percentage). Abbreviations: PNI: prognostic nutritional index; HBV: hepatitis B virus; HCV: hepatitis C virus; APRI: aspartate aminotransferase to platelet ratio index; ALT: alanine aminotransferase; INR: international normalized ratio; AFP: alpha-fetoprotein.

**Table 7:** Comparison of characteristics between patients with high and low SII.

	High SII (>160, *n* = 559)	Low SII (≤160, *n* = 144)	*p* Value
Male Sex	452 (80.9%)	101 (70.1%)	0.006
Age, Years	58.87 ± 11.66	58.41 ± 11.46	0.672
HBV Infection	316 (56.5%)	81 (56.3%)	1.000
HCV Infection	191 (34.2%)	60 (41.7%)	0.098
APRI	0.84 ± 1.60	1.32 ± 1.00	0.001
ALT, U/L	53.62 ± 91.86	56.50 ± 39.24	0.714
Neutrophil, /μL	4193.03 ± 2970.90	2004.19 ± 611.43	<0.001
Lymphocyte, /μL	1713.61 ± 651.65	1977.01 ± 780.13	<0.001
Platelet, ×10^3^/μL	171.02 ± 80.96	115.46 ± 41.09	<0.001
Total bilirubin, mg/dL	0.80 ± 0.34	0.81 ± 0.30	0.822
Albumin, g/dL	3.62 ± 0.61	3.50 ± 0.60	0.054
Prothrombin Time, INR	1.03 ± 0.07	1.06 ± 0.10	<0.001
Moderate/Poor Differentiation	480 (87.9%)	131 (91.0%)	0.377
Microvascular Invasion	211 (53.0%)	67 (65.0%)	0.034
AFP, μg/L	516.49 ± 2293.49	873.22 ± 4145.54	0.175

Data are expressed as mean ± standard deviation or number (percentage). Abbreviations: SII: systemic immune-inflammation index; HBV: hepatitis B virus; HCV: hepatitis C virus; APRI: aspartate aminotransferase to platelet ratio index; ALT: alanine aminotransferase; INR: international normalized ratio; AFP: alpha-fetoprotein.

## Abbreviations

HCC	hepatocellular carcinoma
NLR	neutrophil-to-lymphocyte ratio
PLR	platelet to lymphocyte ratio
PNI	prognostic nutritional index
SII	systemic immune-inflammation index
